# Global discovery of small RNAs in the fish pathogen *Edwardsiella piscicida*: key regulator of adversity and pathogenicity

**DOI:** 10.1186/s13567-018-0613-z

**Published:** 2018-12-11

**Authors:** He-he Du, Hai-Zhen Zhou, Ping Tang, Hui-qin Huang, Min Liu, Yong-hua Hu

**Affiliations:** 1 Institute of Tropical Bioscience and Biotechnology, Key Laboratory of Biology and Genetic Resources of Tropical Crops of Ministry of Agriculture, Chinese Academy of Tropical Agricultural Sciences, Haikou, 571101 China; 2 Laboratory for Marine Biology and Biotechnology, Qingdao National Laboratory for Marine Science and Technology, Qingdao, China; 3 Hainan Provincial Key Laboratory for Functional Components Research and Utilization of Marine Bio-resources, Haikou, 571101 China; 4 Key Laboratory of Experimental Marine Biology, Institute of Oceanology, Chinese Academy of Sciences, Qingdao, 266071 China; 5 University of Chinese Academy of Sciences, Beijing, 100049 China; 6 Yunnan Agricultural University, Kunming, Yunnan 650200 China

## Abstract

Recently, bacterial small RNA (sRNA) has been shown to be involved as a key regulator in stress responses. sRNAs of *Edwardsiella piscicida*, an important aquatic pathogen, are not well characterized to date. In this study, using RNA-seq technology, we globally found and identified sRNA candidates expressed from *E. piscicida* grown in normal LB medium, acid pressure, iron deficiency stress, and oxidation pressure. A total of 148 sRNAs were found, including 19 previously annotated sRNAs and 129 novel sRNA candidates by searching against the Rfam database. Compared in normal condition, the expression of 103 sRNAs (DEsRNA, differentially expressed sRNA) and 1615 mRNAs (DEmRNAs, differentially expressed mRNA) showed significant differences in three stress sample. Based on the prediction by IntaRNA and relational analysis between DEsRNAs and DEmRNAs, 103 DEsRNAs were predicted to regulate 769 target mRNAs. Pleiotropic function of target DEmRNAs indicated that sRNAs extensively participated in a variety of physiological processes, including response to adversity and pathogenicity, the latter was further confirmed by infection experiment. A large number transcription factors appeared in target genes of sRNAs, which suggested that sRNAs likely deeply interlaced within complex gene regulatory networks of *E. piscicida*. Moreover, 49 Hfq-associated sRNAs were also identified in this study. In summary, we globally discovered sRNAs for the first time in pathogenic bacteria of fish, and our findings indicated that sRNAs in *E. piscicida* have important roles in adaptation to environmental stress and pathogenicity. These results also provide clues for deciphering regulation mechanism of gene expression related to physiological response and pathogenicity.

**Electronic supplementary material:**

The online version of this article (10.1186/s13567-018-0613-z) contains supplementary material, which is available to authorized users.

## Introduction

*Edwardsiella piscicida* (formerly included in *E. tarda*) [1, 2], one family member of Enterobacteriaceae, is a Gram-negative, motile, rod-shaped bacterium. It is a serious fish pathogen and infects a wide range of host that includes multiple species of economically important fish such as Japanese flounder (*Paralichthys olivaceus*), turbot (*Scophtalmus maximus*), eel (*Anguilla japonica*), striped bass (*Morone saxatilis*), red sea bream (*Pagrus major*), tilapia (*Oreochromis niloticus*), and channel catfish *(Ictalurus punctatus*) [3–5]. Fish infected by *E. piscicida* frequently develop a systemic disease called edwardsiellosis, which in Japanese flounder is often manifested in hemorrhage, septicemia, skin lesions, and necrosis of liver, gut, and kidney [4, 6]. Heavy economic losses due to *E. piscicida*-related edwardsiellosis have been reported to occur in the Asia, United States, and Europe. Currently, control of *E. piscicida* in aquaculture relies chiefly on antibiotics in most countries including China. In recent years, a large number of studies have been carried out to examine the virulence mechanism of *E. piscicida* in different fish models. Many virulence factors/systems, such as type III (T3SS) and type VI (T6SS) secretion systems, two-component regulatory system, hemolysin, LuxS/AI-2 quorum sensing system, molecular chaperons and RNA-binding protein Hfq, ferric uptake regulator, lysozyme inhibitors, and so on, are known to be involved in *E. piscicida* stress resistance or pathogenicity [7–16]. However, the regulation of stress resistance and pathogenicity in *E. piscicida* is largely unknown so far.

Bacteria are constantly exposed to stressful and challenging environment. To cope with adverse environment and to survive, bacteria have evolved intricate mechanisms to sense the surrounding milieu and to adequately respond by changing their gene expression patterns and thus phenotypes [17]. Among various regulatory factors, small RNA (sRNA) of bacteria have attracted more and more attention in recent years. The majority of sRNAs function as regulators of gene expression at the post-transcriptional level and play critical regulatory roles in major biological processes, such as adaptation to various environmental stresses, quorum sensing, biofilm formation, motility, and pathogenicity [17–24]. Bacterial sRNAs are usually untranslated transcripts with length ranges from 50 to 500 nucleotides. Almost all of the so far characterized sRNAs regulate their target mRNA’s translation and/or stability by the way of base pairing [25]. Binding of sRNA with ribosome-binding sites (RBSs) blocks initiating ribosomes and then inhibit translation initiation. As positive regulators, they can either stabilize the mRNA by interfering cleavage of RNase or enhance translation by sRNA-binding-induced structure modulation to render translation initiation sites available [17, 26, 27]. Bacterial sRNA can be divided into two types: *cis*-encoded and *trans*-encoded sRNA. The former is expressed from the same locus as their sole target with which they share full complementarity. The latter is expressed from loci elsewhere sharing only partial complementarity with their targets and target multiple mRNAs with specific seed sequences [28, 29]. In many bacteria, *trans*-encoded sRNAs often need the help of Hfq, a close relative of the Sm/Lsm family of proteins involved in splicing and RNA decay. The important homohexameric helper protein has multiple effects on mRNA translation by protection and stabilization of sRNAs from degradation, by facilitating its interaction with the target mRNA, or by increasing the rate of sRNA–mRNA annealing [17, 23, 30]. On the contrary, *cis*-encoded sRNAs do not require Hfq for stability and regulation. Deleting Hfq, which has pleiotropic effects on the stability of several sRNAs, results in numerous phenotypes, including resistance to environmental stresses and pathogenicity [31, 32]. Our previous study showed that Hfq played an important role in responding to adversity and pathogenicity of *E. piscicida* [13], but its mechanism remains unknown.

Currently, the study about sRNA in the pathogenic bacteria of teleost fish was very scarce. Only several sRNAs were identified in *E. tarda* [33]. Information about the number and function of sRNAs in *E. piscicida* is unknown. In this present study, we discovered and identified sRNAs in *E. piscicida* globally and analyzed their functions in the pathogenicity of *E. piscicida*.

## Materials and methods

### Bacteria and growth conditions

*Edwardsiella piscicida* TX01 was isolated from diseased fish [34]. *Escherichia coli* DH5α and S17-1λpir were purchased from Tiangen (Beijing, China) and Biomedal (Sevilla, Spain), respectively. Bacteria were cultured in Luria–Bertani broth (LB) at 37 °C (for *E. coli*) or 28 °C (for *E. piscicida*). Where indicated, 2,2′-dipyridyl (DP), chloramphenicol, and polymyxin B were supplemented at the concentration of 100 μM, 30 μg/mL, and 100 μg/mL, respectively.

*Edwardsiella piscicida* TX01 was cultured in normal LB medium or stress condition, i.e., in LB medium with pH = 5.0 (acid stress, Ac), in LB medium with 100 μM dipyridyl (iron deficiency, Dp), and in LB medium with 500 μM hydrogen peroxide (oxidation pressure, Pe). Bacteria were cultured to exponential phase and collected. Then the bacteria were used for subsequent RNA sequencing. The experiment was performed three times.

### RNA isolation and RNA sequencing

sRNA isolation, library construction, and high-throughput sequencing were carried out by Beijing Genomics Institute (BGI), Shenzhen, China. Total RNA was isolated using TRIzol Reagent according to the manufacturer’s instructions (Invitrogen). The RNA samples were treated with DNaseI to remove residual genomic DNA. The quantity and purity of the RNA were monitored using a NanoDrop™ 1000 spectrophotometer and agarose gel electrophoresis. RNA fragments with length of 50 nt to 500 nt were isolated following gel filtration and purification. A total of four small RNA libraries were constructed and single-end sequencing was performed on an Illumina Hiseq 4000 by BGI. At the same time, the total RNA libraries of four samples were prepared and sequenced.

### Analysis of sequencing reads

The reads were processed by filtering low quality reads, removing adapter and impurity sequence, then the clean reads were obtained. After assembling the clean reads, the full-length tags were obtained. The tags were mapped with genome of *E. piscicida*, which produce bioinformatics of sequence alignment. Among these information, the tags within intergenic or intragenic region of genes were chosen to the candidate sRNAs. Analysis of the follow-up biological information of these candidate sRNAs included: (1) sRNA annotation, (2) prediction of promoter and Rho-independent transcription terminators, (3) prediction of secondary structure, (4) prediction of target genes by IntaRNA, (5) GO annotation of target genes, (6) differential expression analysis of sRNA and mRNA. The relative transcript abundance was measured by reads per kilobase of transcript per million (RPKM) mapped sequence reads. The differential expression analysis of sRNA and mRNA was performed using the DEGseq package. The sequences of sRNAs were extracted and searched against the sRNAMap, Rfam database, sRNATarBase, SIPHI, and BSRD.

### Northern blot analysis

Northern blot analysis was carried out using a DIG Northern Starter Kit (Roche) following the manufacturer’s protocol as described by Yan et al. [35].

### Quantitative real-time reverse transcription-PCR (qRT-PCR)

qRT-PCR were carried out as reported previously [14].

### sRNAs knockout

The primers used in this study were listed in Table 1. To construct the sRNA knockout mutant, in-frame deletion of segment of sRNA was performed by overlap extension PCR as follows: the first overlap PCR was performed with the primer pair KOF1 and KOR1, the second overlap PCR was performed with the primer pair KOF2 and KOR2, and the fusion PCR was performed with the primer pair KOF1 and KOR2. The PCR products were inserted into the suicide plasmid pDM4 at the BglII site, resulting in pDMsRNA. S17-1λpir was transformed with pDMsRNA, and the transformants were conjugated with TX01 as described previously [13]. The transconjugants were selected on LB agar plates supplemented with 10% sucrose. One of the colonies that were resistant to sucrose and sensitive to chloramphenicol (marker of pDM4) was analyzed by PCR, and the PCR products were subjected to DNA sequencing to confirm in-frame deletion.

**Table 1:** **Primers used in this study**

Primer	Sequences (5′ → 3′)
sR012KOF1	GGATCCAGTCCCTCTCTTCGCA (*Bam*HI)
sR012KOR1	AGGCAAGTTACGACGCAAGTATTGCA
sR012KOF2	GCGTCGTACTTGCCTGTCGGCAGGT
sR012KOR2	GGATCCGCGCAGCAAATCGTCGT (*Bam*HI)
sR043KOF1	GGATCCTCGAGCCGTGAACTGTT (*Bam*HI)
sR043KOR1	ATAGATCGCTGCGTAAAAAATGCGCA
sR043KOF2	TTACGCAGCGATCTATTTGGTGAATGGT
sR043KOR2	GGATCCATTCAAACTCGCTCAGGT (*Bam*HI)
sR082KOF1	GGATCCGCCTTTGCTCCAGATAAT (*Bam*HI)
sR082KOR1	CAAATACCCGGAAAAGCCCATACAAT
sR082KOF2	CTTTTCCGGGTATTTGCTGGCCTATCCT
sR082KOR2	GGATCCAGCCACTGAATAGCGAAG (*Bam*HI)
SR084KOF1	GGATCCTGGAACGAGATCGAGAT (*Bam*HI)
SR084KOR1	ACGCAAAAAGCAATGGGGATATTGTCT
SR084KOF2	CCATTGCTTTTTGCGTCCGCGTTCT
SR084KOR2	GGATCCATCCCGATACCCGACAA (*Bam*HI)
SR114KOF1	GGATCCGGTGTCGTGGCTTGAAGT (*Bam*HI)
SR114KOR1	TTTACCAAAGCTCATTGCCTATTTGGAT
SR114KOF2	AATGAGCTTTGGTAAACAGGTGGTGTTT
SR114KOR2	GGATCCCACAAACCCAGCAAGCGCT (*Bam*HI)
SR145KOF1	GGATCCCTGCGACCTTGTCCGTT (*Bam*HI)
SR145KOR1	CGCACAGAACGCTATTCTGACGCATT
SR145KOF2	AATAGCGTTCTGTGCGACATGTCGTT
SR145KOR2	GGATCCTGGATTTCGAACTCTACGT (*Bam*HI)
SR274KOF1	GGATCCTCATCCGTAAATGGGTGAT (*Bam*HI)
SR274KOR1	CCGAAAAGGCAATCAGCGCTACGCAT
SR274KOF2	CTGATTGCCTTTTCGGCCTGGTTCT
SR274KOR2	GGATCCCACAGGAAGGGCGAT (*Bam*HI)
SR318KOF1	GGATCCCTGGTAGGTCGTGCCA (*Bam*HI)
SR318KOR1	AATGAGCCATCCCCTGTCGTCTTCCT
SR318KOF2	CAGGGGATGGCTCATTATCTCACAGGCA
SR318KOR2	GGATCCCTTCTACCTGGCGCTGAT (*Bam*HI)
SR355KOF1	GGATCCTGTGGCGGTGCAGCATT (*Bam*HI)
SR355KOR1	TCGCACTTGAACGTGCACCGGCCT
SR355KOF2	GCACGTTCAAGTGCGAGCCGCGCAAT
SR355KOR2	GGATCCGTCATGCGCAGGGTTT (*Bam*HI)

### Identification of Hfq-associated sRNAs

To identify the Hfq-associated sRNAs, a markerless *hfq* in-frame deletion mutant TXhfq [13], was used this study. After culturing to exponential phase, TX01 and TXhfq were collected and RNA isolation were conducted as describe as above. sRNAs expressions were analyzed by RNA sequencing.

### Cellular infection and pathogenicity analysis

FG-9307 cells were cultured at 23 °C in 96-well cell culture plates (~10^5^ cells/well) with L-15 medium (Gibco, USA) as described previously [36]. The cells were infected with TX01 or sRNA mutants at a MOI of 10:1 for 2 h. After washing with PBS three times, the cells were lysed with 1% Triton X-100, and the lysate was plated on LB agar plates. After incubation at 28 °C for 24 h, the colony number was counted.

### Statistical analysis

All statistical analyses were performed with SPSS 18.0 software (SPSS Inc., Chicago, IL, USA). Data were analyzed with analysis of variance (ANOVA), and statistical significance was defined as *P* < 0.05.

## Results

### Sequencing of RNA from *E*. *piscicida*

To identify globally sRNAs and investigate the sRNA transcriptome profiles of *E. piscicida*, total RNA was separately isolated from bacteria grown in normal media (Con) and three adverse conditions, i.e., acidic condition (Ac), iron deficiency (Dp), and oxidation pressure (Pe). Small-fragment RNAs with length of 50 to 500 nt were gel-purified and subjected to deep sequencing. Removing reads of poor quality and N > 10%, a total of 26 765 798 reads was obtained. After splicing, a total of 1 622 257 unique tags from four samples were mapped to *E. piscicida* genome. After removing tRNAs, rRNAs, and repeated tags, 5663 tags as candidate sRNA were produced.

### Discovery of the sRNAs in *E*. *piscicida*

We extracted sRNA transcripts with sequential bases forming peaks of high-level expression which are distinct from those of its flanking regions. A total of 148 sRNAs with predicted promoter or Rho-independent terminator were finally identified (Additional file 1). Against the Rfam database and sRNAMap, 19 annotated sRNA homologs were detected. The remaining 129 sRNAs appeared to be novel sRNA candidates. The most highly expressed sRNAs (> 10 000 RPKM in four samples) include sR176, sR318, sR100, sR065 (RsmY), sR111, sR214. The expression of 26 sRNAs distribute among 1000–9999 RPKM in four samples.

### Validation and characterization of selected sRNA candidates

**Figure 1:**
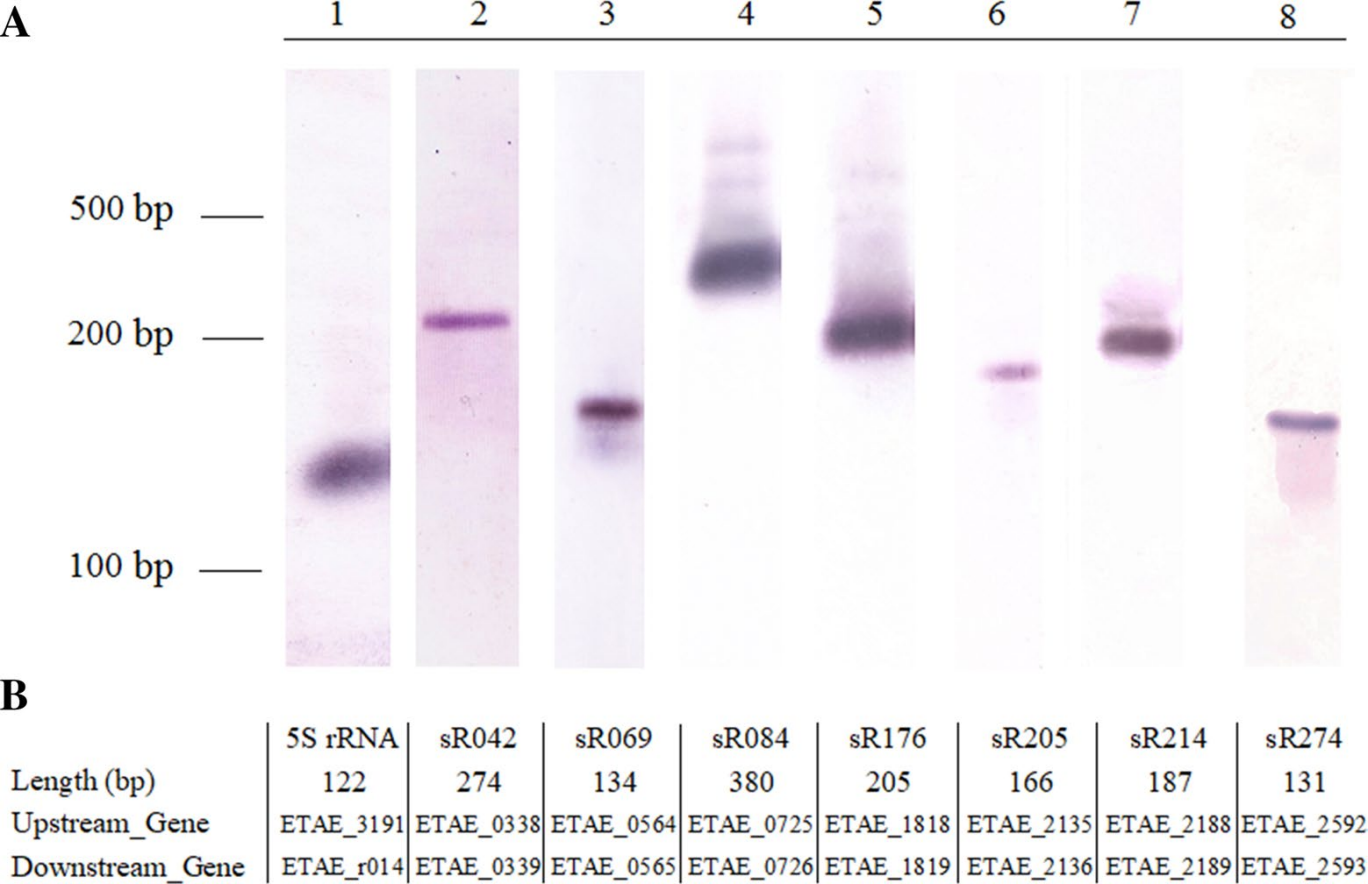
**Validation of selected sRNA candidates by Northern blot analysis. A** *Edwardsiella piscicida* TX01 were cultured in normal LB medium, RNA was isolated and used to Northern blot analysis. 5S rRNA as positive control. **B** The length, upstream gene, and downstream gene of RNA.

To check whether the boundaries of sRNAs transcripts were in accordance with those predicted by our methods, the sizes of one annotated sRNA (sR084) and six novel sRNAs (sR042, sR069, sR176, sR205, sR214, and sR274) were determined by Northern blot, and 5S rRNA as a control. The results showed that the transcript lengths of seven sRNAs detected by Northern blot analysis were approximately consistent with the lengths observed by deep sequencing (Figure 1).

### sRNAs expression in different conditions

**Figure 2:**
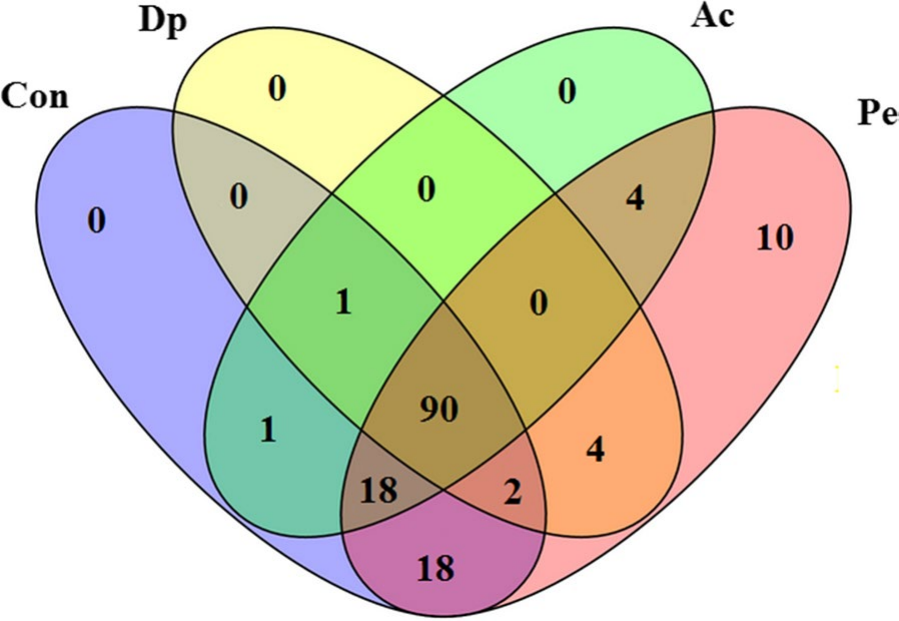
**Venn diagram of sRNA expression during four different conditions.** *Edwardsiella piscicida* TX01 was culture in normal LB medium (Con), in LB medium with pH = 5.0 (acid stress, Ac), in LB medium with 100 μM dipyridyl (iron deficiency, Dp), and in LB medium with 500 μM hydrogen peroxide (oxidation pressure, Pe). The numbers inside the diagram stand for the numbers of sRNA.

The expression profiles of the 148 sRNAs were normalized with RPKM and then tested for significant difference among different samples with Bonferroni correction. The results showed that 90 sRNAs were expressed in all examined sample (Con, Ac, Dp, and Pe) (Figure 2). Ten sRNAs were specifically expressed in Pe sample. One, eighteen, four, and four sRNAs were expressed in Con and Ac, Con and Pe, Dp and Pe, and Ac and Pe, respectively. One sRNA was expressed in three sample of Con, Ac, and Dp. Two sRNAs were expressed in Con, Dp, and Pe. Eighteen sRNAs were expressed in Con, Pe, and Ac. No sRNA was specifically expressed in Con, Ac, or Dp. No sRNA was specifically expressed in Con and Dp, Dp and Ac, Dp and Ac and Pe (Figure 2).

### Differentially expressed sRNAs (DEsRNAs) during stress condition

**Figure 3:**
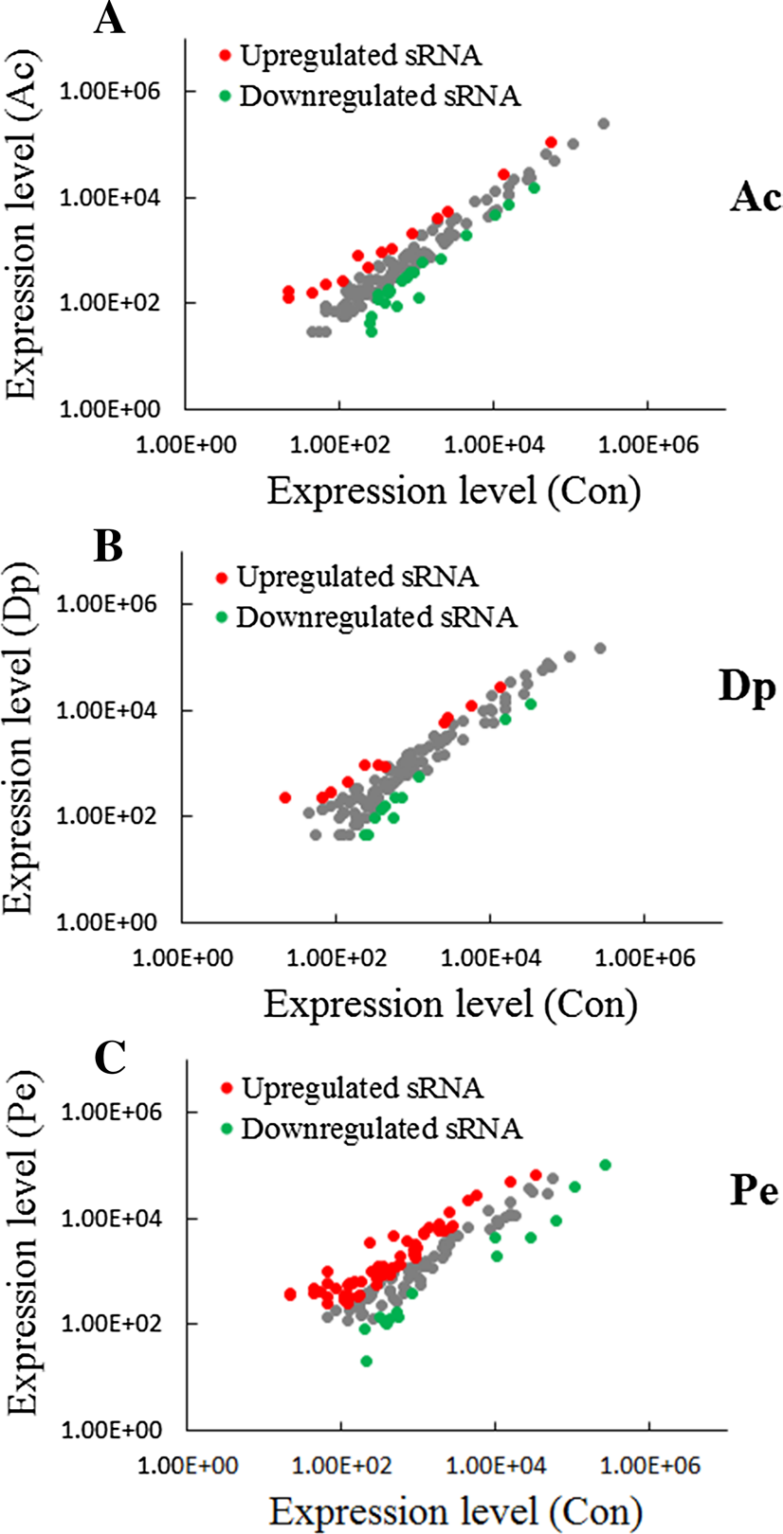
**Differential expression of sRNAs during three stress condition.** Scatter plot of the sRNA expression levels in acid stress (Ac) (**A**), iron deficiency (Dp) (**B**), and oxidation pressure (Pe) (**C**) in comparison with that in normal LB medium (Con). Red and green spots represent sRNAs significantly upregulated and downregulated, respectively.

Compared to Con, 103 sRNAs showed significant differences (> twofold and *P* < 0.05) in three stress sample (Ac, Dp, and Pe) (Figure 3 and Additional file 2). For convenience, these differentially expressed sRNAs were named DEsRNAs. Facing acid pressure (Ac), 15 sRNAs were significantly upregulated and 26 sRNAs were significantly downregulated. In iron deficiency (Dp), 13 sRNAs were significantly upregulated and 13 sRNAs were significantly downregulated. When bacteria grew in medium with hydrogen peroxide (Pe), 67 sRNAs were significantly upregulated and 15 sRNAs were significantly downregulated. There are 15, 20, 19 sRNAs showed significant differences in both Ac and Dp, both Dp and Pe, and both Ac and Pe, respectively. Ten sRNAs expressions showed significant differences in all three adverse environments, most of them were downregulated. Specifically, four sRNAs (sR162, sR165, sR188, and sR231) were consistently downregulated and two sRNAs (sR230 and sR355) were consistently upregulated, four sRNAs (sR040, sR214, sR281, sR365) were downregulated in Ac and Dp but upregulated in Pe (Figure 3 and Additional file 2).

### Differentially expressed mRNAs (DEmRNA) during stress condition

**Figure 4:**
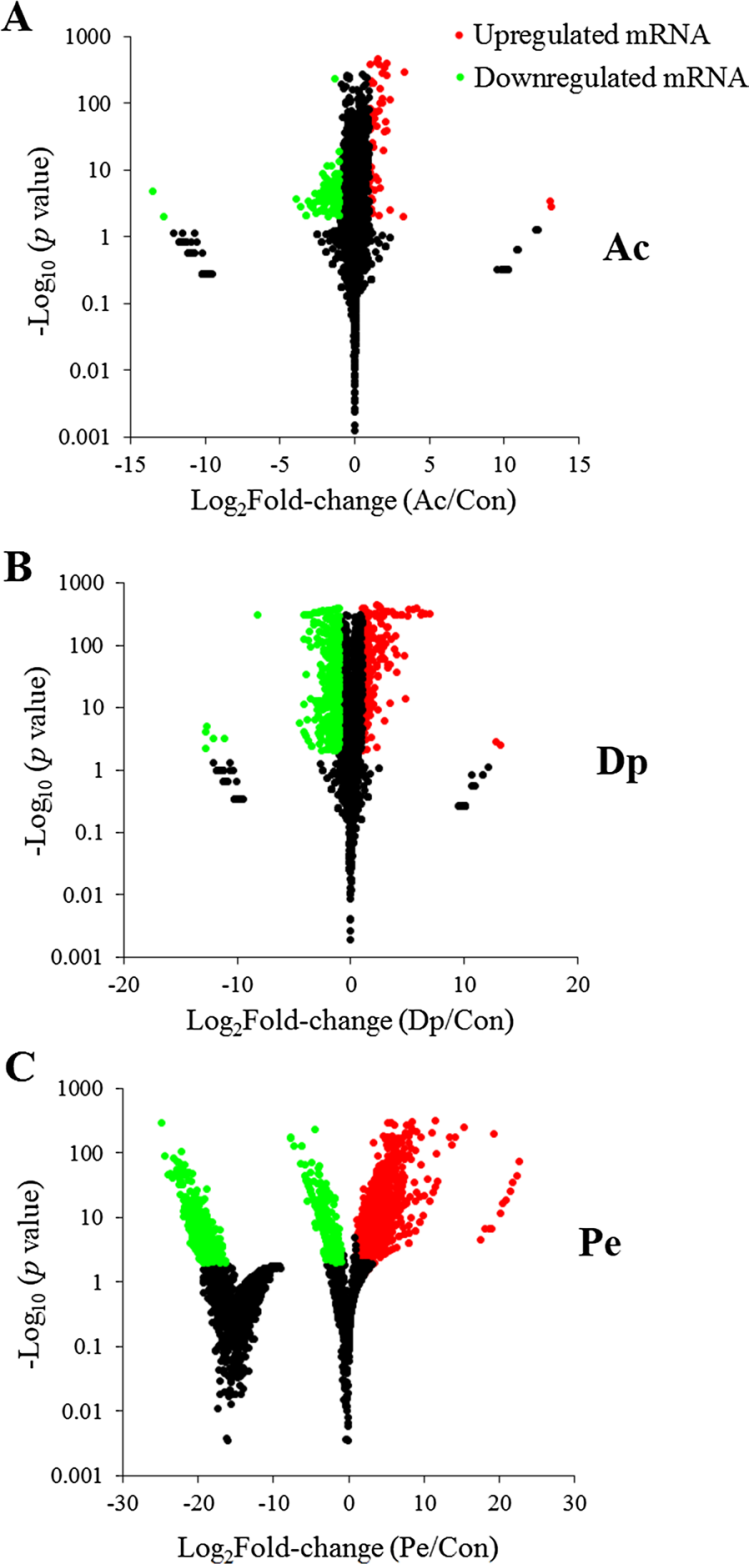
**Effect of stress on the expression of** ***Edwardsiella piscicida*** **genes.** MA-plots show differentially expressed genes in acid stress (Ac) (**A**), iron deficiency (Dp) (**B**), and oxidation pressure (Pe) (**C**) in comparison with that in normal LB medium (Con). Red and green spots represent genes significantly upregulated and downregulated, respectively.

In order to investigate the effect of the sRNA on target gene, global change in gene expressions associated with stress condition was examined. Based on the RNA-Seq data, it was found that the expressions of 1615 genes were significantly (> twofold and *P* < 0.01) altered during stress condition (Figure 4). Compared to Con, 55 genes expressions were significantly upregulated and 99 genes were significantly downregulated in Ac, 278 genes were significantly upregulated and 408 genes were significantly downregulated in Dp, 916 genes were significantly upregulated and 626 genes were significantly downregulated in Pe (Additional file 3). For convenience, these differentially expressed mRNAs were named DEmRNAs. Of these DEmRNAs, 20 DEmRNAs were existed in all three sample, 29, 16, and 198 DEmRNAs were found in Ac and Dp, Ac and Pe, and Dp and Pe, respectively (Additional file 3).

### Prediction of DEsRNA target genes among DEmRNA

Based on the prediction of DEsRNA target genes by IntaRNA and relational analysis between DEsRNA and DEmRNA, 103 DEsRNAs were predicted to regulate 769 target mRNAs (Additional file 4). Of these sRNAs, sR225 have only one predicted target mRNA. However, sR226 was predicted 19 target mRNAs. Of 769 target mRNAs, LysR family transcriptional regulator (ETAE_0919) was predicted to be regulated by 15 sRNAs. A total of 31 transcriptional regulator were predicted to be regulated by 45 sRNAs, forming 60 sRNA–mRNA pairs (Additional file 4). These transcriptional regulators include a variety of types, such as GntR family, TetR family, AraC family, ArsR family, DeoR family, GntR family, LysR family, LuxR family, AHL-dependent regulator, and two-component transcriptional regulator. A total of 68 hypothetical proteins were predicted to be regulated by 96 sRNAs, forming 282 sRNA–mRNA pairs (Additional file 4). In addition, there are 48 sRNA–mRNA pairs involved in transporter, 14 sRNA–mRNA pairs involved in acid/cold/heat shock protein, 32 sRNA–mRNA pairs involved in iron/ferredoxin/ferrous/hemin/hemagglutinin, 13 sRNA–mRNA pairs involved in type III/VI secretion system, and 4 sRNA–mRNA pairs involved in universal stress protein.

### Enrichment analysis of DEsRNA target genes

**Figure 5:**
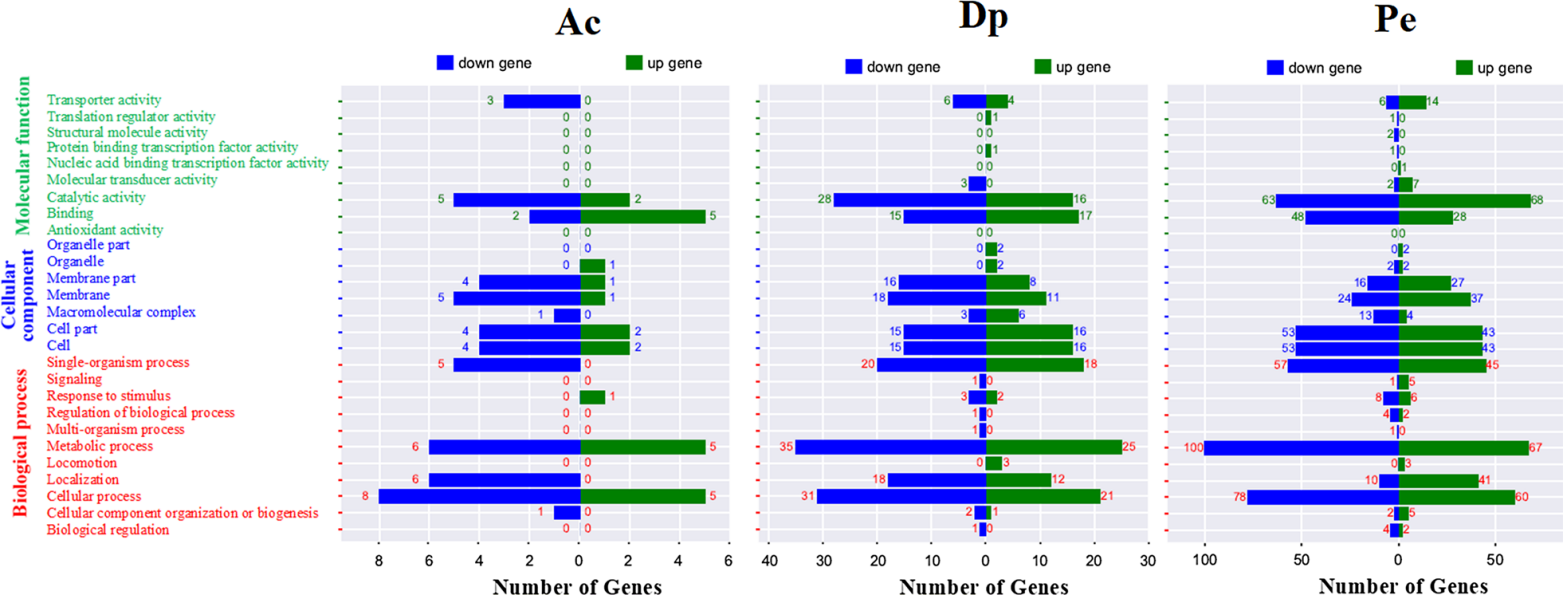
**Gene ontology categories of differentially expressed genes in acid stress (Ac), iron deficiency (Dp), and oxidation pressure (Pe) based on Go analysis.** Green: molecular function, blue: cellular component, red: biological process.

The DEsRNA target genes identified above were subjected to gene ontology (GO) analysis. As shown in Figure 5, GO annotation of the 769 target genes indicated that they were grouped into three major categories: Biological process, Cellular component, and Molecular function. In the group of “Biological process”, the largest number of genes belong to metabolic process term. In the group of “Cellular Component”, the term of cell and cell part make up the main part. In the group of “Molecular function”, the largest number of genes is catalytic activity term. In particular, some genes involved acid tolerance were observed among the target genes, including genes coding acid shock protein, putrescine transport protein PotE, NADH dehydrogenase, and succinate dehydrogenase. Iron uptake and transport related genes, such as genes coding siderophore biosynthesis protein, hemin transport, hemin uptake protein, hemin receptor, ion transport protein, and ferrous iron transport protein, were among the target genes. Genes participating response to oxidation pressure, such as superoxide dismutase SodB, fumarate reductase, cytochrome D ubiquinol oxidase, methionine-R-sulfoxide reductase, and NADH:ubiquinone oxidoreductase appeared in the target genes. Moreover, there are a large number of genes that play an important role in virulence among these DEsRNA target genes, for example, heat shock protein, molecular chaperone, invasion, adhesin, methyl-accepting chemotaxis protein, regulator of cell autolysis, temperature sensitive hemagglutinin, type III secretion, type VI secretion system protein, and so on. Target genes of sRNAs appear versatile.

### Hfq-associated sRNAs

**Figure 6:**
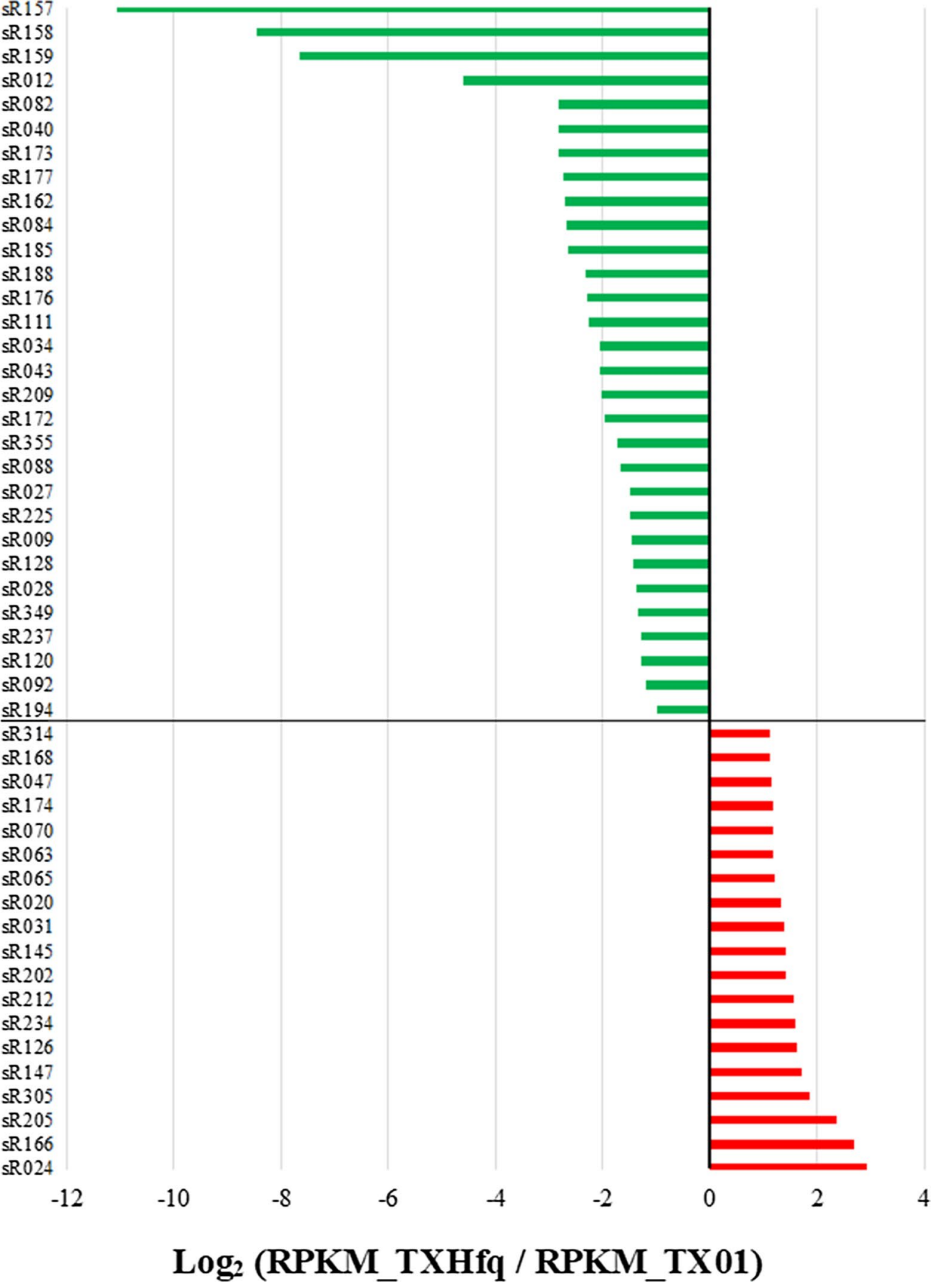
**Identification of Hfq-dependent sRNAs.** *Edwardsiella piscicida* TX01 and TXhfq, a markerless *hfq* in-frame deletion mutant were collected and RNA isolation were conducted. sRNA expression was analyzed by RNA sequencing. sRNAs showed significant difference expression (> two fold and *P* < 0.05) were identified to Hfq-dependent sRNA.

Since Hfq is an important RNA chaperone protein, many sRNAs are Hfq-dependent [32]. Our previous study showed that Hfq mutation attenuated remarkably bacterial virulence [13], which suggested Hfq-associated sRNAs maybe play an important role in pathogenicity of *E. piscicida*. To identify the Hfq-associated sRNAs, the different expression of sRNAs between TX01 and TXhfq, a markerless *hfq* in-frame deletion wild type, was analyzed by RNA-seq. The results showed that 19 sRNAs expression were significantly upregulated and 30 sRNAs expressions were significantly downregulated when Hfq was inactivated (Figure 6). qRT-PCR was conducted to examine the mRNA levels of 5 upregulated sRNAs and 14 downregulated sRNAs. The results showed that, of 5 upregulated sRNA identified by RNA-seq, 4 sRNAs were significantly upregulated by qRT-PCR, of 14 downregulated sRNAs identified by RNA-seq, 12 sRNAs expression were significantly downregulated or undetectable by qRT-PCR (Additional file 4).

### sRNAs potentially involved in *E. piscicida* pathogenicity

**Figure 7:**
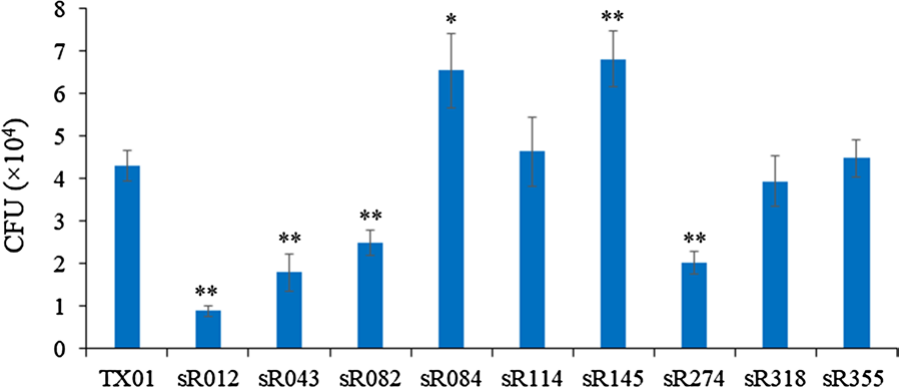
**Pathogenicity analysis of sRNA mutants.** FG-9307 cells were infected with TX01 or sRNA mutants. At 2 h post-infection, bacterial numbers were determined by plate count. The experiment was performed three times, and values are shown as mean ± SEM. *P < 0.05, **P < 0.01.

Since sRNAs widely participate in bacterial pathogenicity, we investigated the potential roles of different kinds of sRNAs in *E. piscicida* pathogenicity. Six Hfq-associated sRNAs (sR012, sR043, sR082, sR084, sR145, and sR355) were chosen to perform the pathogenicity experiment. Meanwhile, three Hfq-nonassociated sRNAs, such as high expression sRNA (sR318, see Additional file 5), moderate expression sRNA (sR274), and low expression sRNA (sR114), were also included. These nine sRNAs mutants were constructed and their pathogenicity were examined. The results showed that the ability of four sRNAs to infect host cell declined significantly, and the ability of two sRNAs to infect host cell enhanced significantly, compared to wild strain TX01 (Figure 7). Among the six Hfq-associated sRNAs, only one sRNA (sR355) was not related to pathogenicity. Among the three Hfq-nonassociated sRNAs, only one sRNA (sR274) was related to pathogenicity.

## Discussion

During the last decade, sRNAs have emerged as essential post-transcriptional regulators in bacteria. Nearly all important physiological and stress responses are modulated by these sRNAs regulator. Researchers have successfully predicted, identified, and characterized sRNAs in a large number Gram-negative and Gram-positive species. It is assumed that an average bacterial genome encodes about 100–300 sRNAs [20]. Currently, sRNAs in many human pathogen and plant pathogen, such as *Yersinia pestis*, *Yersinia pseudotuberculosis*, *Streptococcus pneumoniae*, and *Agrobacterium tumefaciens*, have been identified [35, 37–39]. In this study, 148 candidate sRNAs in fish pathogen *E. piscicida* were identified, including 19 annotated sRNA homologs. As far as we know, our study is the first report about systematic identification of sRNA in fish pathogen.

Homohexameric RNA chaperone Hfq has been shown to play a critical role in sRNA-mediated gene regulation. In many bacteria, besides playing a role in protection of sRNAs from RNase E-mediated degradation, Hfq has been considered to a key factor in sRNA-mediated gene regulation and efficient base pairing between trans-encoded sRNA and its target mRNA [40, 41]. For example, CyaR and RprA interaction with their targe mRNA required Hfq [42]. Deleting Hfq predictably resulted in numerous phenotypes, mainly consisting of resistance to various environmental stresses [31, 43]. In our previous report, we found that deletion of Hfq in *E. piscicida* exhibited multiple effects, including retarding retarded planktonic and biofilm growth, decreased resistance against oxidative stress, and attenuated virulence, and Hfq exerted a regulatory effect on a wide range of genes [13]. In this study, deficiency of Hfq influenced the expression of 49 sRNAs, of which 30 sRNAs expressions were downregulated, several sRNAs expression even could not be detected in *hfq* mutant by qRT-PCR. These results indicated that a great many of sRNAs were Hfq-associated sRNA in *E. piscicida*, and the regulatory effect of Hfq on target genes were likely achieved by sRNAs.

In their natural habitats, bacteria are constantly exposed to stressful and even challenging conditions. Studies have shown that bacterial sRNAs play important roles as regulators in coping with stress and survival. For example, sRNA DsrA and RprA both confer acid resistance [44]. RyhB is a key actor of iron homeostasis regulation [45]. MicF and OxyS are related to oxidative stress [46, 47]. In this study, during acid condition, 41 sRNA expressions were significantly affected, and upregulated sRNAs were more than downregulated sRNA among the DEsRNAs expressions. Especially, sRNA004, sRNA050, sRNA040, and sRNA371 displayed extremely remarkable difference expression. These findings suggested that multiple sRNAs in *E. piscicida* deeply participated in resisting to acid pressure. sRNA040 is homologous with sraG, which was reported to participates in PNPase homeostasis [48], but there is no report of sraG about acid tolerance to the present. During iron deficiency stress, the expressions of 26 sRNAs changed significantly in *E. piscicida*, the amount of upregulated sRNAs were basically equal to those of downregulated sRNA among the DEsRNAs expressions. These DEsRNAs include some annotated sRNA such as RyhB (sR355) and CyaR (sR103), and many novel sRNA such as sRNA043 and sRNA300. It is well known that RyhB was involved in regulating iron homeostasis [45]. It was reported that CyaR in *E. coli* participated in regulating an acid-resistance membrane protein HdeD [42], and its regulation of target genes assisted bacterial survival in the face of envelope stress [49]. However, in *E. piscicida*, we observed that the expression of CyaR was regulated by iron deficiency stress, but not by acid stress. Unlike the expression of sRNA in acid stress and iron deficiency stress, when facing with the stress of hydrogen peroxide, more sRNAs (82 sRNAs) expression were affected, and the vast majority of sRNAs (about 3/4) exhibited upregulated expression upon oxidative stress. These DEsRNAs included some annotated sRNA such as GlmZ_SraJ, rhyA, csrB, csrC, and a lot novel sRNA such as sR073, sR147, and sR370. Amongst the three different kinds of adversity stress, the sum of DEsRNAs, and the number of upregulated sRNA or downregulated sRNA showed distinct difference, which indicated that sRNAs possessed diversity of function. Facing adversity stress, many significantly upregulated sRNAs have target mRNAs that were downregulated. On the other hand, some downregulated sRNAs have upregulated target genes. These results indicated that sRNAs could potentially negatively regulate target genes, which was similar to other studies [50].

Except as an important regulator of adaptation to adversity, sRNAs also play a key role in bacterial pathogenesis. The role of bacterial sRNAs in virulence has received more and more attention in recent years. It was reported that many of the identified sRNAs in human pathogen *S. pneumoniae* have important global and niche-specific roles in virulence [38]. In many other pathogens, a large number sRNAs, such as Rli27, LhrC, teg49, RsmY, AsdA, were reported to participate in regulation of virulence [51–53]. Lately, several sRNAs were identified in *E. tarda* and speculated to play a regulatory role of virulence [33]. Consistently, in this study, a large number of target genes of sRNAs were virulence-associated genes, such as temperature sensitive hemagglutinin, invasion, heat shock protein, cell autolysis factor, type III secretion system protein, type VI secretion system protein, and so on. And we also examined the role of several sRNAs in virulence. Of the nine sRNAs used to detect pathogenicity, 6 sRNAs were found to involve in infection of host FG cell. It is known *E. piscicida* can invade and survive in FG-9307 cells, which often used as an in vitro infection model of *E. piscicida* [54]. However, in vitro cell infections are different from in vivo animal infection and partially address the pathogenicity. In vivo animal experiments are needed to confirm the role of these sRNAs in the pathogenesis of the infection.

As regulator, the function of sRNA is reflected by its target genes. In *E. piscicida*, we found that sRNAs could target a large number transcription factors, including LysR family transcriptional regulator, GntR family transcriptional regulator, two-component transcriptional regulator, phage transcriptional regulatory, transcriptional repressor, transcriptional activator, and so on. These discoveries provided obvious evidence that sRNA likely deeply interlaced within complex gene regulatory networks of *E. piscicida*. Except transcriptional regulator, various types of functional target genes of sRNAs appeared. For example, *dps*, which was involved in iron limitation and acid stress [55], showed differentially expression in acid and oxidation pressure and was predicted to be regulated by sR031. *atoE*, which might be required for the resistance to prolonged acid exposure [56], displayed downregulated expression in acid stress and was predicted to be regulated by sR084 and sR177. During iron deficiency, the target genes of sRNAs included many iron uptake and transport genes, such as genes encoding ferrous iron transport protein A, iron transporter, periplasmic ferric iron-binding protein, ABC transporter. In our previous study, we found Fur showed a very close relationship with iron homeostasis in *E. piscicida*, and regulated a lot of proteins including PotE, SpeF, SpeF2, Ndh, ompF, and napA, which play important role in helping bacterial adapt adverse circumstance [14]. Similarly, in this study, these genes were found to be among targets of sRNAs and showed differently expression in iron deficiency environment. Compared the acid stress and iron deficiency, much more sRNA target genes were found in oxidation pressure condition. A lot of genes directly related to oxidation stress, such as gene encoding alkyl hydroperoxide reductase, thiol peroxidase [57], chaperonin GroEL and GroES [58], superoxide dismutase SodB and SodC [14, 59], thioredoxin TrxA and TrxH [60], were among target DEmRNAs. Moreover, many mRNA targets of DEsRNAs code hypothetical proteins, including some very significant differentially expressed hypothetical proteins, which indicate that many unknown things remain to be elucidated in this organism.

In conclusion, we globally discovered candidate sRNAs for the first time in pathogenic bacteria of fish. Many novel sRNAs were identified and expression patterns of DEsRNAs and DEmRNAs in *E. piscicida* during adversity condition growth were revealed. DEsRNA target genes among DEmRNAs were predicted. The role of sRNAs in *E. piscicida* pathogenicity was characterized. Hfq-associated sRNAs were also identified. Our findings showed that sRNAs in *E. piscicida* have important functions in adaptation to environmental stress and pathogenicity. These results also provide clues for deciphering regulation mechanism of gene expression related to physiological response and pathogenicity in *E. piscicida*.

## Additional files

**Additional file 1.** **The information on 148 sRNAs in** ***Edwardsiella piscicida*** **identified by RNA-seq analysis**.

**Additional file 2.** **Differential expression anlysis of sRNAs during three stress condition.**

**Additional file 3.** **Differentially expressed mRNA.**

**Additional file 4.** **Predicted DEsRNA target genes.**

**Additional file 5.** **The expression of Hfq-dependent sRNAs were identified by quantitative real time PCR.** Five upregulated sRNAs (left) and 14 downregulated sRNAs (right) identified by RNA-sequencing were checked by qRT-PCR. The experiment was performed three times, and values are shown as mean ± SEM. *P < 0.05, **P < 0.01.

## References

[1] Abayneh T, Colquhoun DJ, Sørum H (2013). *Edwardsiella piscicida* sp. nov., a novel species pathogenic to fish. J Appl Microbiol.

[2] Liu Y, Zhao L, Yang M, Yin K, Zhou X, Leung KY, Liu Q, Zhang Y, Wang Q (2017). Transcriptomic dissection of the horizontally acquired response regulator EsrB reveals its global regulatory roles in the physiological adaptation and activation of T3SS and the cognate effector repertoire in *Edwardsiella piscicida* during infection toward turbot. Virulence.

[3] Mohanty BR, Sahoo PK (2007). Edwardsiellosis in fish: a brief review. J Biosci.

[4] Ucko M, Colorni A, Dubytska L, Thune RL (2016). *Edwardsiella piscicida*-like pathogen in cultured grouper. Dis Aquat Organ.

[5] Sun K, Wang HL, Zhang M, Xiao Z, Sun L (2009). Genetic mechanisms of multi-antimicrobial resistance in a pathogenic *Edwardsiella tarda* strain. Aquaculture.

[6] Rashid MM, Nakai T, Muroga K, Miyazaki T (1997). Pathogenesis of experimental edwardsiellosis in Japanese flounder *Paralichthys olivaceus*. Fish Sci.

[7] Cao H, Han F, Tan J, Hou M, Zhang Y, Yang D, Liu Q (2018). *Edwardsiella piscicida* type III secretion system effector EseK inhibits mitogen-activated protein kinase phosphorylation and promotes bacterial colonization in Zebrafish Larvae. Infect Immun.

[8] Cui S, Xiao J, Wang Q, Zhang Y (2018). H–NS binding to evpB and evpC and repressing T6SS expression in fish pathogen *Edwardsiella piscicida*. Arch Microbiol.

[9] Chakraborty S, Sivaraman J, Leung KY, Mok YK (2011). The two-component PhoBPhoR regulatory system and ferric uptake regulator sense phosphate and iron to control virulence genes in type III and VI secretion systems of *Edwardsiella tarda*. J Biol Chem.

[10] Wang X, Wang Q, Xiao J, Liu Q, Wu H, Zhang Y (2010). Hemolysin EthA in *Edwardsiella tarda* is essential for fish invasion in vivo and in vitro and regulated by two-component system EsrA–EsrB and nucleoid protein HhaEt. Fish Shellfish Immunol.

[11] Zhang M, Sun K, Sun L (2008). Regulation of autoinducer 2 production and luxS expression in a pathogenic *Edwardsiella tarda* strain. Microbiology.

[12] Dang W, Hu YH, Sun L (2011). HtpG is involved in the pathogenesis of *Edwardsiella tarda*. Vet Microbiol.

[13] Hu YH, Li YX, Sun L (2014). *Edwardsiella tarda* Hfq: impact on host infection and global protein expression. Vet Res.

[14] Hu YH, Sun L (2016). The global regulatory effect of *Edwardsiella tarda* Fur on iron acquisition, stress resistance, and host infection: a proteomics-based interpretation. J Proteomics.

[15] Li MF, Wang C, Sun L (2015). *Edwardsiella tarda* MliC, a lysozyme inhibitor that participates in pathogenesis in a manner that parallels Ivy. Infect Immun.

[16] Srinivasa Rao PS, Lim TM, Leung KY (2001). Opsonized virulent *Edwardsiella tarda* strains are able to adhere to and survive and replicate within fish phagocytes but fail to stimulate reactive oxygen intermediates. Infect Immun.

[17] Holmqvist E, Wagner EGH (2017). Impact of bacterial sRNAs in stress responses. Biochem Soc Trans.

[18] Waters LS, Storz G (2009). Regulatory RNAs in bacteria. Cell.

[19] Updegrove TB, Zhang A, Storz G (2016). Hfq: the flexible RNA matchmaker. Curr Opin Microbiol.

[20] Gimpel M, Brantl S (2017). Dual-function small regulatory RNAs in bacteria. Mol Microbiol.

[21] Gripenland J, Netterling S, Loh E, Tiensuu T, Toledo-Arana A, Johansson J (2010). RNAs: regulators of bacterial virulence. Nat Rev Microbiol.

[22] Papenfort K, Vogel J (2014). Small RNA functions in carbon metabolism and virulence of enteric pathogens. Front Cell Infect Microbiol.

[23] Bossi L, Figueroa-Bossi N (2016). Competing endogenous RNAs: a target-centric view of small RNA regulation in bacteria. Nat Rev Microbiol.

[24] Dutta T, Srivastava S (2018). Small RNA-mediated regulation in bacteria: a growing palette of diverse mechanisms. Gene.

[25] Storz G, Vogel J, Wassarman KM (2011). Regulation by small RNAs in bacteria: expanding frontiers. Mol Cell.

[26] Papenfort K, Vanderpool CK (2015). Target activation by regulatory RNAs in bacteria. FEMS Microbiol Rev.

[27] Nitzan M, Rehani R, Margalit H (2017). Integration of bacterial small RNAs in regulatory networks. Annu Rev Biophys.

[28] Papenfort K, Bouvier M, Mika F, Sharma CM, Vogel J (2010). Evidence for an autonomous 50 target recognition domain in an Hfq-associated small RNA. Proc Natl Acad Sci U S A.

[29] Balbontín R, Fiorini F, Figueroa-Bossi N, Casadesús J, Bossi L (2010). Recognition of heptameric seed sequence underlies multi-target regulation by RybB small RNA in *Salmonella enterica*. Mol Microbiol.

[30] De Lay N, Schu DJ, Gottesman S (2013). Bacterial small RNA-based negative regulation: Hfq and its accomplices. J Biol Chem.

[31] Chao Y, Vogel J (2010). The role of Hfq in bacterial pathogens. Curr Opin Microbiol.

[32] Kavita K, de Mets F, Gottesman S (2018). New aspects of RNA-based regulation by Hfq and its partner sRNAs. Curr Opin Microbiol.

[33] Sun Y, Zhang J, Qin L, Yan C, Zhang X, Liu D (2017). Identification and validation of sRNAs in *Edwardsiella tarda* S08. PLoS One.

[34] Cheng S, Zhang M, Sun L (2010). The iron-cofactored superoxide dismutase of *Edwardsiella tarda* inhibits macrophage-mediated innate immune response. Fish Shellfish Immunol.

[35] Yan Y, Su S, Meng X, Ji X, Qu Y, Liu Z, Wang X, Cui Y, Deng Z, Zhou D, Jiang W, Yang R, Han Y (2013). Determination of sRNA expressions by RNA-seq in *Yersinia pestis* grown in vitro and during infection. PLoS One.

[36] Zhou ZJ, Zhang L, Sun L (2015). *Pseudomonas fluorescens*: fur is required for multiple biological properties associated with pathogenesis. Vet Microbiol.

[37] Koo JT, Alleyne TM, Schiano CA, Jafari N, Lathem WW (2011). Global discovery of small RNAs in *Yersinia pseudotuberculosis* identifies Yersinia-specific small, noncoding RNAs required for virulence. Proc Natl Acad Sci U S A.

[38] Mann B, van Opijnen T, Wang J, Obert C, Wang YD, Carter R, McGoldrick DJ, Ridout G, Camilli A, Tuomanen EI, Rosch JW (2012). Control of virulence by small RNAs in *Streptococcus pneumoniae*. PLoS Pathog.

[39] Lee K, Wang K (2018). Small noncoding RNAs in *Agrobacterium tumefaciens*. Curr Top Microbiol Immunol.

[40] Brennan RG, Link TM (2007). Hfq structure, function and ligand binding. Curr Opin Microbiol.

[41] Azam MS, Vanderpool CK (2018). Translational regulation by bacterial small RNAs via an unusual Hfq-dependent mechanism. Nucleic Acids Res.

[42] Lalaouna D, Prévost K, Laliberté G, Houé V, Massé E (2018). Contrasting silencing mechanisms of the same target mRNA by two regulatory RNAs in *Escherichia coli*. Nucleic Acids Res.

[43] Christiansen JK, Larsen MH, Ingmer H, Sogaard-Andersen L, Kallipolitis BH (2004). The RNA-binding protein Hfq of *Listeria monocytogenes*: role in stress tolerance and virulence. J Bacteriol.

[44] Bak G, Han K, Kim D, Lee Y (2014). Roles of *rpoS*-activating small RNAs in pathways leading to acid resistance of *Escherichia coli*. Microbiologyopen.

[45] Meibom KL, Cabello EM, Bernier-Latmani R (2018). The small RNA RyhB is a regulator of cytochrome expression in *Shewanella oneidensis*. Front Microbiol.

[46] Delihas N, Forst S (2001). Micf: an antisense RNA gene involved in response of *Escherichia coli* to global stress factors. J Mol Biol.

[47] Altuvia S, Weinstein-Fischer D, Zhang A, Postow L, Storz G (1997). A small, stable RNA induced by oxidative stress: role as a pleiotropic regulator and antimutator. Cell.

[48] Fontaine F, Gasiorowski E, Gracia C, Ballouche M, Caillet J, Marchais A, Hajnsdorf E (2016). The small RNA SraG participates in PNPase homeostasis. RNA.

[49] Vogt SL, Evans AD, Guest RL, Raivio TL (2014). The Cpx envelope stress response regulates and is regulated by small noncoding RNAs. J Bacteriol.

[50] Gelsinger DR, DiRuggiero J (2018). Transcriptional landscape and regulatory roles of small non-coding RNAs in the oxidative stress response of the haloarchaeon *Haloferax volcanii*. J Bacteriol.

[51] Ortega AD, Quereda JJ, Pucciarelli MG, García-del Portillo F (2014). Non-coding RNA regulation in pathogenic bacteria located inside eukaryotic cells. Front Cell Infect Microbiol.

[52] Manna AC, Kim S, Cengher L, Corvaglia A, Leo S, Francois P, Cheung AL (2018). Small RNA teg49 is derived from a sarA transcript and regulates virulence genes independent of SarA in *Staphylococcus aureus*. Infect Immun.

[53] Oliva G, Sahr T, Buchrieser C (2015). Small RNAs, 5′UTR elements and RNA-binding proteins in intracellular bacteria: impact on metabolism and virulence. FEMS Microbiol Rev.

[54] Hu YH, Zhou HZ, Jin QW, Zhang J (2016). The serine protease autotransporter Tsh contributes to the virulence of *Edwardsiella tarda*. Vet Microbiol.

[55] Yu MJ, Ren J, Zeng YL, Zhou SN, Lu YJ (2009). The *Legionella pneumophila* Dps homolog is regulated by iron and involved in multiple stress tolerance. J Basic Microbiol.

[56] Dong Q, Hyde D, Herra C, Kean C, Murphy P, O’Morain CA, Buckley M (2001). Identification of genes regulated by prolonged acid exposure in *Helicobacter pylori*. FEMS Microbiol Lett.

[57] Hajaj B, Yesilkaya H, Shafeeq S, Zhi X, Benisty R, Tchalah S, Kuipers OP, Porat N (2017). CodY regulates thiol peroxidase expression as part of the pneumococcal defense mechanism against H_2_O_2_ stress. Front Cell Infect Microbiol.

[58] Santra M, Dill KA, de Graff AMR (2018). How do chaperones protect a cell’s proteins from oxidative damage. Cell Syst.

[59] Oh E, McMullen L, Jeon B (2015). Impact of oxidative stress defense on bacterial survival and morphological change in *Campylobacter jejuni* under aerobic conditions. Front Microbiol.

[60] Reott MA, Parker AC, Rocha ER, Smith CJ (2009). Thioredoxins in redox maintenance and survival during oxidative stress of *Bacteroides fragilis*. J Bacteriol.

