# Supplementary figures and images for: Global discovery of small RNAs in the fish pathogen Edwardsiella piscicida: key regulator of adversity and pathogenicity
Source: Vet Res. 2018 Dec 11;49:120. doi: 10.1186/s13567-018-0613-z (PMC6288947; doi:10.1186/s13567-018-0613-z)

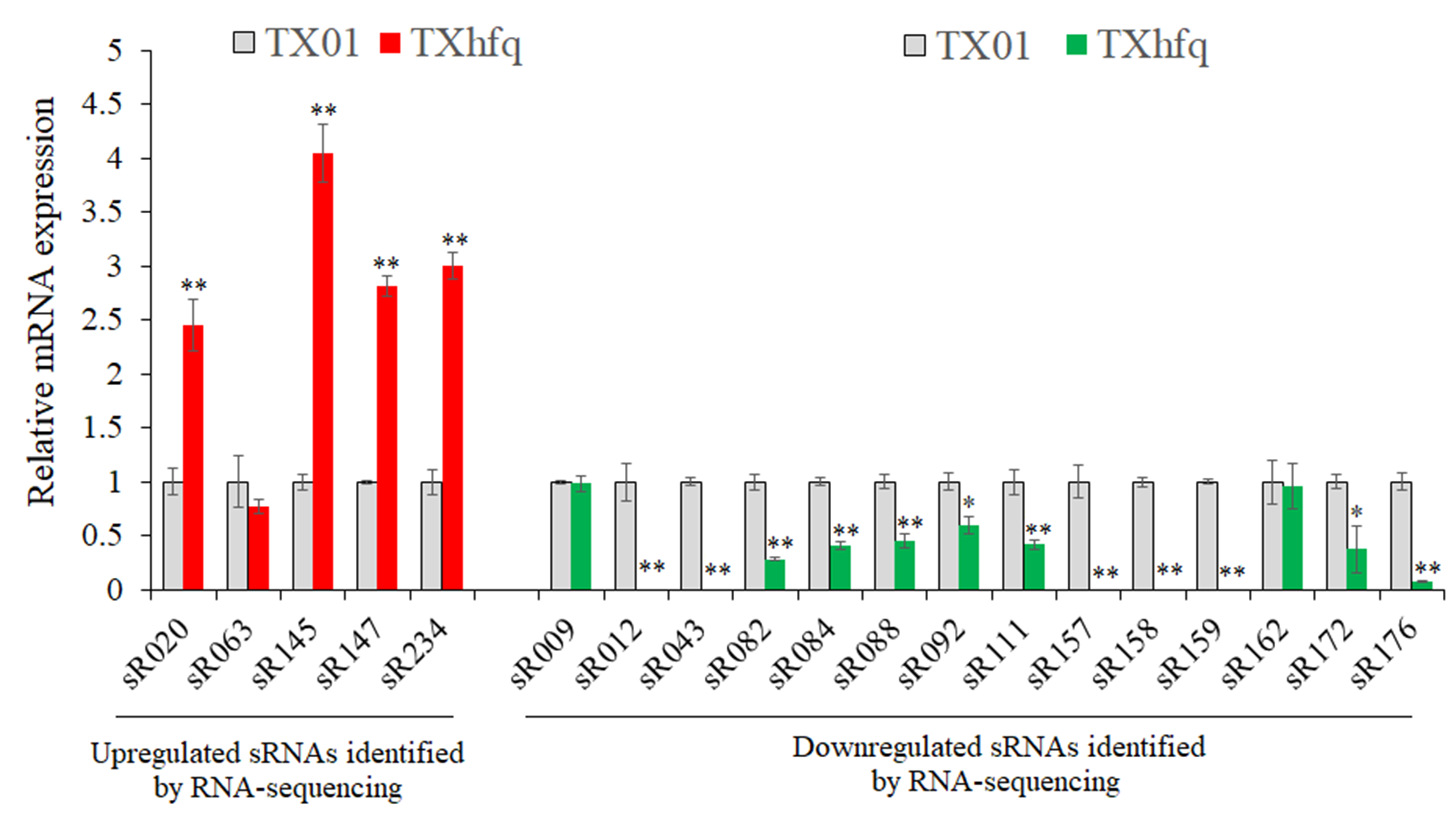

Supplement: Supplementary file 1 — Additional file 1. The information on 148 sRNAs in Edwardsiella piscicida identified by RNA-seq analysis. [file 13567_2018_613_MOESM1_ESM.tif]
